# Control of Polyamine Biosynthesis by Antizyme Inhibitor 1 Is Important for Transcriptional Regulation of Arginine Vasopressin in the Male Rat Hypothalamus

**DOI:** 10.1210/en.2015-1074

**Published:** 2015-05-11

**Authors:** Michael P. Greenwood, Mingkwan Greenwood, Julian F. R. Paton, David Murphy

**Affiliations:** School of Clinical Sciences (M.P.G., M.G., D.M.), University of Bristol, Bristol BS1 3NY, United Kingdom; School of Physiology and Pharmacology (J.F.R.P.), University of Bristol, Bristol BS8 1TD, United Kingdom; and Department of Physiology (D.M.), University of Malaya, Kuala Lumpur, Malaysia 50603

## Abstract

The polyamines spermidine and spermine are small cations present in all living cells. In the brain, these cations are particularly abundant in the neurons of the paraventricular (PVN) and supraoptic nuclei (SON) of the hypothalamus, which synthesize the neuropeptide hormones arginine vasopressin (AVP) and oxytocin. We recently reported increased mRNA expression of antizyme inhibitor 1 (*Azin1*), an important regulator of polyamine synthesis, in rat SON and PVN as a consequence of 3 days of dehydration. Here we show that AZIN1 protein is highly expressed in both AVP- and oxytocin-positive magnocellular neurons of the SON and PVN together with antizyme 1 (AZ1), ornithine decarboxylase, and polyamines. *Azin1* mRNA expression increased in the SON and PVN as a consequence of dehydration, salt loading, and acute hypertonic stress. In organotypic hypothalamic cultures, addition of the irreversible ornithine decarboxylase inhibitor DL-2-(difluoromethyl)-ornithine hydrochloride significantly increased the abundance of heteronuclear AVP but not heteronuclear oxytocin. To identify the function of *Azin1* in vivo, lentiviral vectors that either overexpress or knock down *Azin1* were stereotaxically delivered into the SON and/or PVN. *Azin1* short hairpin RNA delivery resulted in decreased plasma osmolality and had a significant effect on food intake. The expression of AVP mRNA was also significantly increased in the SON by *Azin1* short hairpin RNA. In contrast, *Azin1* overexpression in the SON decreased AVP mRNA expression. We have therefore identified AZIN1, and hence by inference, polyamines as novel regulators of the expression of the AVP gene.

Polyamines play important roles in many physiological processes, with documented effects on gene expression, protein function, regulation of ion channels, and hormone secretion (1). Vertebrates have two polyamines, spermidine and spermine. Their formation is catalyzed by the conversion of ornithine to putrescine by ornithine decarboxylase (ODC; encoded by the *Odc* gene), the initial and rate-limiting enzyme of polyamine biosynthesis (2). The activity of ODC, and thus the ability of the cell to synthesize polyamines, is controlled by the relative abundance of antizyme 1 (AZ1; encoded by the *Az1* gene) that binds to ODC and targets it for proteolytic degradation (3). However, this system is subject to additional regulation by ODC-related proteins termed antizyme inhibitors (encoded by the *Azin1* and *Azin2* genes), which possess high affinity for AZ1 to rescue ODC from its complex with AZ1, thereby neutralizing AZ1 functions (4).

Throughout development, high levels of polyamines have been reported in the brain and other tissues. These levels decline into adulthood (5). For secretory cells, high levels of polyamines persist in adult animals (6). Interestingly, studies mapping spermidine/spermine distribution throughout the adult rat brain identified strong neuronal staining of these polyamines in the magnocellular neurons (MCNs) of the paraventricular nucleus (PVN) and supraoptic nucleus (SON) of the hypothalamo-neurohypophyseal system (HNS) (7, 8).

The HNS is the source of arginine vasopressin (AVP). Synthesized in cell bodies of the large MCNs of the SON and PVN, AVP is transported anterogradely to terminals in the posterior pituitary gland (9). A rise in plasma osmolality is detected by intrinsic MCN osmoreceptor mechanisms (10) and osmoreceptive neurones in the circumventricular organs that project to (11, 12) and provide direct excitatory inputs that increase the firing of MCNs (13), resulting in hormone secretion (14). AVP travels through the bloodstream to the kidney in which it promotes water and sodium reabsorption (15). The HNS also produces the closely related hormone oxytocin (OT) from a separate population of MCNs (16), which acts to promote kidney natriuresis (17).

Dehydration (DH) evokes a dramatic functional remodeling of the HNS (18), which might contribute to the facilitation of hormone production and delivery. Microarrays have been used to ask how DH evokes changes in the rat HNS transcriptome that may mediate these plastic events (19, 20). One of the genes discovered to be up-regulated in the HNS as a consequence of DH was *Azin1*. We thus hypothesized that antizyme inhibitor 1 (AZIN1) was perhaps important in regulating AVP and OT biosynthesis. In this study we document ODC, AZ1, and AZIN1 expression in AVP- and OT-positive neurons of the SON and the PVN and identify differential regulation after physiological manipulations. Furthermore, using in vitro and in vivo models to manipulate *Azin1* expression, we have produced evidence of the importance of polyamines for AVP transcriptional regulation.

## Materials and Methods

### Animals

Male Sprague Dawley rats (Harlan Laboratories) were housed in the animal facilities at the University of Bristol. Rats of 250–300 g were maintained under a 14-hour light, 10-hour dark cycle with food and water ad libitum for at least 1 week prior to experimentation. To induce hyperosmotic stress, water was removed (DH) for 1 or 3 days or replaced by 2% (wt/vol) NaCl in drinking water for 1 or 7 days [salt loading (SL)]. In some instances water was returned after DH and SL for 24 hours. The acute responses to elevated plasma osmolality were assessed (10 min, 30 min, 1 h, 2 h, and 4 h) after a single ip injection of 1.5 mL per 100 g body weight of 1.5 M NaCl solution [hypertonic saline (HS)]. The control group had access to food and water ad libitum throughout the experimental period. After injection of HS, the rats were placed back in their home cages and water was removed for the duration of the experiment. Female Sprague Dawley rats with litters of 10 pups (postnatal d 4–5) (Harlan Laboratories) were maintained within the animal facilities for 2 days. Animal experiments were performed between 9:00 am and 2:00 pm. All experiments were performed under a Home Office UK license held under, and in strict accordance with, the provision of the UK Animals (Scientific Procedures) Act (1986) and approval by the University of Bristol Ethical Review Committee.

### Immunofluorescence

Perfusions were performed as described previously (21). For spermine/spermidine immunostaining, rats were perfused with 50 mL of 0.1 M 2-morpholinoethanesulfonic acid monohydrate (Sigma) (pH 5.4), 200 mL 0.1 M 1-(3-dimethyl-aminopropyl)-3-ethylcarbodiimide hydrochloride (Sigma) (pH 5.4), 1000 ml 0.1 M sodium phosphate buffer, and 1000 mL 4% (wt/vol) paraformaldehyde in 0.1 M sodium phosphate buffer. Coronal brain sections (40 μm) were cut on a cryostat, washed in 0.1 M PBS (pH 7.4), and blocked for 30 minutes in 5% (vol/vol) goat serum in 0.1 M PBS containing 0.25% (vol/vol) Triton X-100 (PBST). Sections were incubated with 1:100 dilutions of mouse anti-AZIN1 (Abnova), rabbit anti-ODC (Biomol International), rabbit anti-AZ1 (Biomol International), rabbit antispermine/spermidine (Abcam), mouse anti-OT neurophysin-I (NP-I) [PS38, (22)], guinea pig anti-OT (Peninsula Laboratories), mouse anti-AVP (NP-II, PS41), or rabbit anti-AVP (Sigma) prepared in 1% (vol/vol) goat serum in 0.1 M PBST at 4ºC overnight. Sections were washed and incubated with 1:500 dilution of appropriate biotinylated secondary antibody in PBST for 1 hour at room temperature. Sections were washed and incubated with Alexa Fluor 488 streptavidin-conjugated and Alexa Fluor 594 goat antimouse, rabbit or guinea pig. Sections were mounted and sealed with VectorShields mounting media (Vector Laboratories) (see Supplemental Figure 1 for validation of antibody specificity).

### Real-time quantitative PCR analysis

A 1-mm micropunch (Fine Scientific Tools) was used to collect SON and PVN samples from 60 μm coronal sections in a cryostat and RNA extraction and cDNA synthesis was performed as previously described (21). Primers for *Azin1* (5′-CCGTTATCTCACGGCGAACT-3′ and 5′-CTAGGTTCCCAAGGTGGCTC-3′), *Azin2* (5′-GACGGGGCTTGTGTGTTGCAT-3′ and 5′-CCCAGGTCGGCCACGAAGAA-3′), *Odc* (5′-GTTGCCACATTGACCGTGAC-3′ and 5′-GTTGCCACATTGACCGTGAC-3′), *Az1* (5′-TGTACTCCGACGAGCGGCTG-3′ and 5′-GTGACCTGCTTGGCCTCCGT-3′), *c-Fos* (5′-AGCATGGGCTCCCCTGTCA-3′ and 5′-GAGACCAGAGTGGGCTGCA-3′), heteronuclear AVP (hnAVP) (5′-GAGGCAAGAGGGCCACATC-3′ and 5′-CTCTCCTAGCCCATGACCCTT-3′), mature AVP (5′-TGCCTGCTACTTCCAGAACTGC-3′ and 5′-AGGGGAGACACTGTCTCAGCTC-3′), heteronuclear OT (hnOT) (5′-TGAGCAGGAGGGGGCCTAGC-3′ and 5′-TGCAAGAGAAATGGGTCAGTGGC-3′), mature OT (5′-TGCCCCAGTCTTGCTTGCT-3′ and 5′-TCCAGGTCTAGCGCAGCCC-3′), enhanced green fluorescent protein (5′-ATCATGGCCGACAAGCAGAAGAAC-3′ and 5′-GTACAGCTCGTCCATGCCGAGAGT-3′), ribosomal protein *Rpl19* (5′-GCGTCTGCAGCCATGAGTA-3′ and 5′-TGGCATTGGCGATTTCGTTG-3′), and glyceraldehyde-3-phosphate dehydrogenase (*Gapdh*) (5′-ATGATTCTACCCACGGCAAG-3′ and 5′-CTGGAAGATGGTGATGGGTT-3′) were synthesized by Eurofins MWG Operon. The quantitative PCRs (qPCRs) were carried out in duplicate using SYBR green (Roche) on an ABI 7500 sequence detection system (Applied Biosystems Inc). For relative quantification of gene expression the 2^−ΔΔCT^ method was used (23). The internal control gene used for these analyses were the housekeeping genes *Rpl19* and *Gapdh*. To analyze *Azin1* gene knockdown, PCRs for gel electrophoresis were performed using primers (*Azin1*, 5′-TGAGCGTGGGAGATTGGCTTAT-3′ and 5′-TTGGCTCAGCTGAATGCAAGAG-3′; *Actin*, 5′-CACCCGCGAGTACAACCTTC-3′ and 5′-CCCATACCCACCATCACACC-3′) and TaqDNA polymerase (New England Biolabs).

### Immunoblotting

A 1-mm micropunch (Fine Scientific Tools) was used to collect SON and PVN samples as detailed for RNA extraction. Punch samples were removed from dry ice and vortexed in ice-cold RIPA buffer (25 mM Tris-HCl, pH 7.6; 150 mM NaCl; 1% (vol/vol) Nonidet P-40; 1% (vol/vol) sodium deoxycholate; 0.1% (wt/vol) sodium dodecyl sulfate; 1 mM EDTA) containing protease inhibitors (Sigma; P8340). For total protein extraction, homogenates were centrifuged at 10 000 × *g* for 15 minutes at 4ºC. Protein samples were prepared in 1× Laemmli buffer solution and heated at 95ºC for 10 minutes, and 20 μg/lane (determined by Bio-Rad Protein Assay) of protein was fractionated on sodium dodecyl sulfate polyacrylamide gels. Membranes were incubated in 5% (wt/vol) skimmed milk in Tris-buffered saline (150 mM NaCl; 20 mM Tris-HCl, pH 7.6) with 0.1% Tween 20 for 1 hour, followed by 1–2500 anti-AZIN1, 1–5000 anti-ODC, 1–2000 anti-β TUBULIN mouse monoclonal antibody (Covance), or 1–10 000 anti-GAPDH mouse monoclonal antibody (Santa Cruz Biotechnology) in blocking buffer. After three washes, the membranes were incubated with the appropriate secondary antibody conjugated with horseradish peroxidase for 1 hour. Membranes were washed with Tris-buffered saline with 0.1% Tween 20. Signal was detected using chemiluminescence ECLPlus reagent (GE Healthcare Biosciences). The immunoblots were stripped in Restore Western blot stripping buffer and reprobed to assess the multiple proteins in the same blot.

### Organotypic cultures

Organotypic slices were prepared as described previously (24). Slices (400 μm) were placed onto hydrated Millipore Millicell-CM filter inserts (three slices per insert) in six-well tissue culture plates containing 1.1 mL of culture medium [50% (vol/vol) Eagle basal medium, 25% (vol/vol) horse serum, 25% (vol/vol) Hanks' balanced salt solution, 0.5% (wt/vol) glucose, 1 mM L-glutamine, 25 μg/mL penicillin, 25 μg/mL streptomycin, and 10 ng/mL ciliary neurotrophic factor]. After 10 days, culture medium was replaced with serum-free medium [SFM; neurobasal A medium containing 2% (vol/vol) B27 supplement, 1 mM sodium pyruvate, 0.075% (vol/vol) sodium bicarbonate, 10 mM HEPES, 2 mM L-glutamine, 0.5% (vol/vol) glucose, 25 μg/mL penicillin, 25 μg/mL streptomycin, and 10 ng/mL ciliary neurotrophic factor]. The cultures were incubated at 35ºC in 5% CO_2_-enriched air and medium was replaced every 2 days. Experimental treatments were performed after 4 days in SFM. Slice cultures were incubated with 0.01% dimethylsulfoxide (DMSO; vehicle) or 10 μm forskolin prepared in SFM for 24 hours. For polyamine studies, slices were placed in SFM with no chemical (control), 10 μm putrescine, 5 mM DL-2-(difluoromethyl)-ornithine hydrochloride (DFMO), or 5 mM DFMO and 10 μm putrescine for 48 hours. The inserts were frozen on dry ice in new six-well tissue culture plates, SON and PVN were punched from three slices in a cryostat, and RNA was extracted as described earlier.

### Construction of lentiviruses

Two short hairpin RNAs (shRNAs) targeting rat *Azin1* (shRNA1-GGAACTGGATTTGCTTGTTCC and shRNA2-GGATATTTACTTCCCTGAAGG) were designed using Invitrogen's Blockit shRNA designer with loop structure TTCAAGAGA and cloned into RNA interference expression vector pSilencer 1.0-U6 (Ambion). The shRNAs were amplified along with the U6 promoter (5′-CCTTAATTAAGGCGACTCACTATAGGGCGAATTGGG-3′ and 5′-CCCGCTCGAGCGGCTAGTGGATCCCCCGGGCTG-3′) from pSilencer 1.0-U6 using Phusion high-fidelity DNA polymerase (New England BioLabs) and cloned into compatible restriction sites of lentiviral vector pRRL.SIN.U6. shRNA.CPPT.CMV.GFP.WPRE. A nontargeting shRNA (GAGGCTATGGTCTACGTTAAT) was generated using siRNA Wizard version 3.1 (www.sirnawizard.com/scrambled.php). cDNAs encoding rat *Azin1* and *Odc* were amplified from rat brain cDNA using primers (5′-ACGGGATCCATGAAAGGATTTATTGACGATG-3′ and 5′-ACTCTCGAGCTAAAGAAGCGTTAATGCC-3′ and 5′-CGAGAATTCACCATGGGCAGCTTTAC-3′ and 5′-TAAGGATCCAGGTAAGAGCTACAAGAATG-3′, respectively), digested, and cloned into corresponding restriction sites of lentiviral vector pRRL.SIN.CPPT.CMV.IRES.GFP.WPRE. A lentiviral vector expressing green fluorescent protein (GFP) was used as a control. High-titer lentiviral vectors were propagated as previously reported (21). All viruses in the present study had a titer of greater than 10^9^ transduction units/mL.

### Lentiviral vector gene transfer into PVN and SON

Stereotaxic injections of lentiviral vectors into the PVN and SON were performed as previously described (21). Lentiviral vector tropism was assessed by visualizing GFP expression in perfused tissue. For metabolic measurements, animals were individually housed in standard laboratory cages for 2 weeks before being transferred to metabolic cages (Techniplast) to allow precise daily measures of fluid intake, food intake, and urine output. Measures were performed 3 days before and 7 days after the addition of 2% (wt/vol) NaCl to drinking water. To investigate gene expression after the overexpression of *Azin1* and *Odc* in the SON, rats received bilateral lentiviral injections, in which *Azin1* or *Odc* was administered to one nuclei and GFP into the other and viral expression was allowed to proceed for 2 weeks. Animals were killed by stunning and decapitation. Trunk blood was collected, and brains were snap frozen on dry ice and stored at −80ºC. AVP levels in extracted plasma samples were determined by ELISA (ADI-900-017; Enzo Life Sciences). Osmolality was measured by freezing point depression using a Roebling microosmometer (Camlab).

### Statistical analysis

Statistical differences between two experimental groups were evaluated using independent-sample unpaired Student's *t* tests. One-way ANOVA with Tukey's post hoc test were used to determine the difference between more than two samples with only a single influencing factor. Two-way ANOVA with Bonferroni post hoc test was used to determine interactions between two independent variables on the dependent variable for metabolic data.

## Results

### Profiling *Azin1*, *Odc*, and *Az1* mRNA expression in rat SON and PVN in response to hypertonic stressors

**Figure 1.**
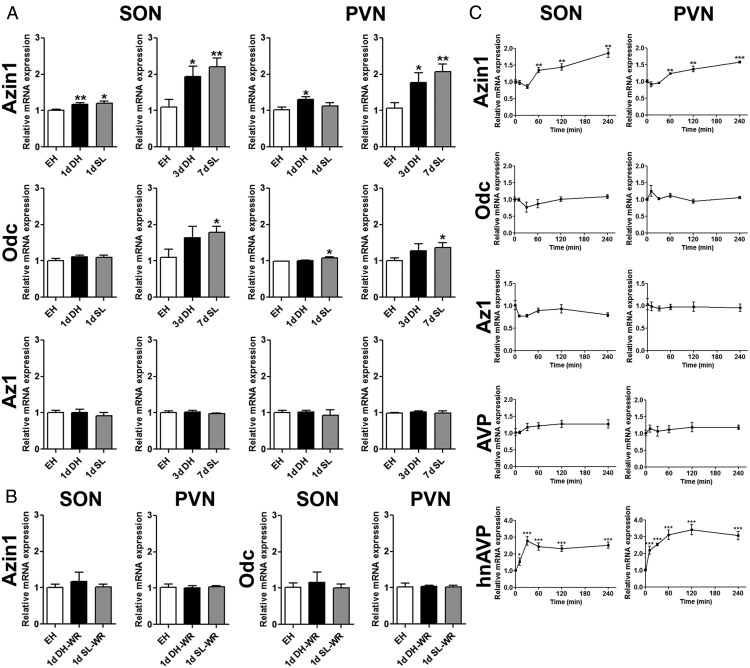
*Azin1*, *Odc*, and *Az1* mRNA expression in rat SON and PVN in response to hyperosmotic stress. The expression of *Azin1*, *Odc*, and *Az1* were examined in the SON and PVN in chronic and acute hyperosmotic conditions and after water repletion. A, Relative mRNA expression of *Azin1*, *Odc*, and *Az1* was investigated by qPCR in SON and PVN of EH, DH (1 and 3 d), and SL (1 and 7 d) rats. B, Relative mRNA of *Azin1* and *Odc* in rat SON and PVN after water repletion for 1 day after 1-day DH and 1-day SL compared with EH controls. C, Changes in *Azin1*, *Odc*, *Az1*, and AVP mRNA expression and hnAVP expression after a single ip injection of 1.5 mL per 100 g body weight 1.5 M NaCl over 240 minutes of the experimental period compared with controls. Values are means ± SEM of n = 5–6 animals per group. *, *P* < .05; **, *P* < .01; ***, *P* < .001.

We used qPCR to investigate the time course of *Azin1*, *Azin2*, *Odc*, and *Az1* mRNA expression in the SON and PVN of euhydrated (EH), DH, and SL rats (Figure 1A). The hnAVP and AVP expression data for SON and PVN has previously been reported (21). The expression of *Azin2* was considerably lower than *Azin1* in SON and PVN of the EH animals, and the expression was unaffected by DH or SL (not shown). In contrast, *Azin1* mRNA expression was significantly higher in SON of 1-day DH (*P* = .008) and 1-day SL (*P* = .02) rats and PVN of 1-day DH rats (*P* = .023) compared with EH controls. Increasing the duration of the hypertonic stress to 3-day DH and 7-day SL further increased the magnitude of this response in both the SON and PVN, with significant increases in *Azin1* mRNA expression in 3-day DH (SON, *P* = .042; PVN, *P* = .038) and 7-day SL (SON, *P* = .006; PVN, *P* = .003) rats compared with EH controls. In contrast to *Azin1*, *Odc* mRNA expression in the SON and PVN of 1-day DH and 1-day SL rats was similar to EH controls, with only PVN from 1-day SL rats (*P* = .025) showing a significantly higher expression. We observed significantly higher *Odc* mRNA expression in SON (*P* = .035) and PVN (*P* = .048) of 7-day SL, but not 3-day DH, rats compared with EH control. No changes in *Az1* mRNA expression were observed in either the SON or PVN after DH or SL at any time point. In a separate experiment, we analyzed the expression of *Azin1* and *Odc* in the SON and PVN of rats in which drinking water was returned for 1-day after either 1-day DH or 1-day SL (Figure 1B). In this instance, there were no differences in *Azin1* or *Odc* mRNA expression compared with EH controls, indicating a quick return to control levels after water repletion.

We then examined the time course of *Azin1*, *Odc*, *Az1*, AVP, and hnAVP accumulation after the acute increase in plasma osmolality resulting from an ip administration of HS. We observed increased *Azin1* mRNA expression in the SON and PVN as early as 60 (SON, *P* = .005; PVN, *P* = .002), 120 (SON, *P* = .004; PVN, *P* = .008), and 240 (SON, *P* = .003; PVN, *P* = 6e^−06^) minutes after injection compared with control, whereas no changes in *Odc* or *Az1* steady-state mRNA levels were evident during the experimental period (Figure 1C). These changes were consistent with significantly increased hnAVP (SON: 10 min, *P* = .047; 30 min, *P* = 1.6e^−04^; 1 h, *P* = 2.2e^−04^; 2 h, *P* = 9.5e^−05^; 4 h, 3.8e^−05^; PVN: 10 min, *P* = .003; 30 min, *P* = 5.6e^−07^; 1 h, *P* = 1.5e^−04^; 2 h, *P* = 8.4e^−05^; 4 h, 1.3e^−04^), but not AVP (Figure 1C), expression in these nuclei resulting from HS administration, perhaps suggesting a modulatory role for *Azin1* in AVP synthesis.

### Colocalization studies of AZIN1, ODC, AZ1, and spermine/spermidine with AVP- or OT-expressing MCNs in the SON and PVN

**Figure 2.**
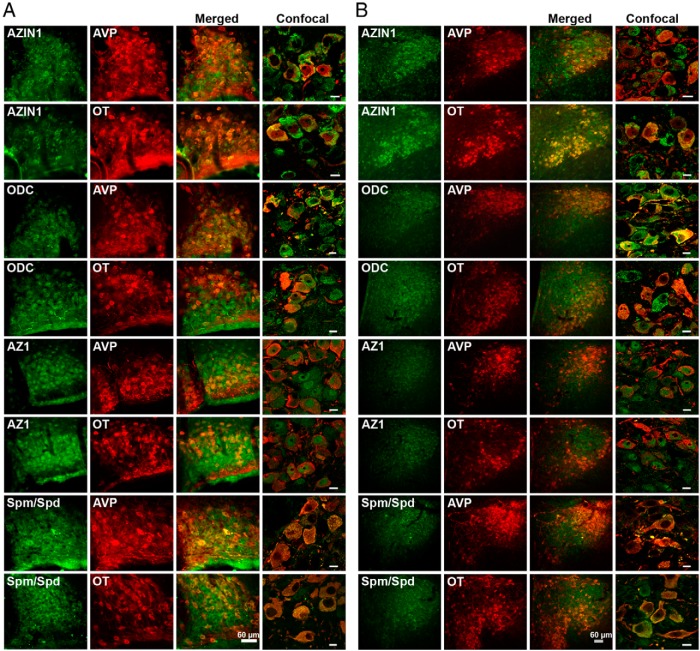
Localization of AZIN1, ODC, AZ1, and spermine/spermidine with AVP and OT neurons of the SON (A) and PVN (B). A and B, Immunofluorescent colocalization of AZIN1, ODC, and AZ1 (all in green) with AVP (red) or OT (red) in the SON and PVN. Confocal images in the right panels show the merged subcellular localization of these proteins and spermine/spermidine with AVP or OT. Spm, spermine; Spd, spermidine. Scale bars for confocal images, 10 μm.

The MCNs of the SON and PVN can be divided into two populations, the OT and AVP MCNs because only a small percentage of MCNs (2%–3%) express high, equivalent levels of both peptides (25). Therefore, to examine the identity of neurons expressing AZIN1, ODC, AZ1, and spermine/spermidine in the SON and PVN of EH rats, double-immunofluorescent staining with AVP NP-II or OT NP-I was performed (Figure 2). AZIN1, ODC, AZ1, and spermine/spermidine staining was observed in all AVP- and OT-expressing neurons in the SON (Figure 2A) and PVN (Figure 2B). Higher-magnification confocal images highlighted the different subcellular localizations of these proteins and spermine/spermidine (Figure 2, A and B). Although AZIN1 protein was predominantly localized in the cell cytoplasm, ODC protein is seen in the cytoplasm and nucleus, whereas the AZ1 protein localizes to the nucleus, with weaker cytoplasmic staining. Spermine/spermidine is found in the cytoplasm (Figure 2). No differences in the subcellular distribution of these proteins or of spermine/spermidine were observed when comparing expression profiles in AVP- or OT-expressing MCNs.

### Expression of AZIN1 and ODC protein in the hypothalamus of EH and 3-day DH rats

**Figure 3.**
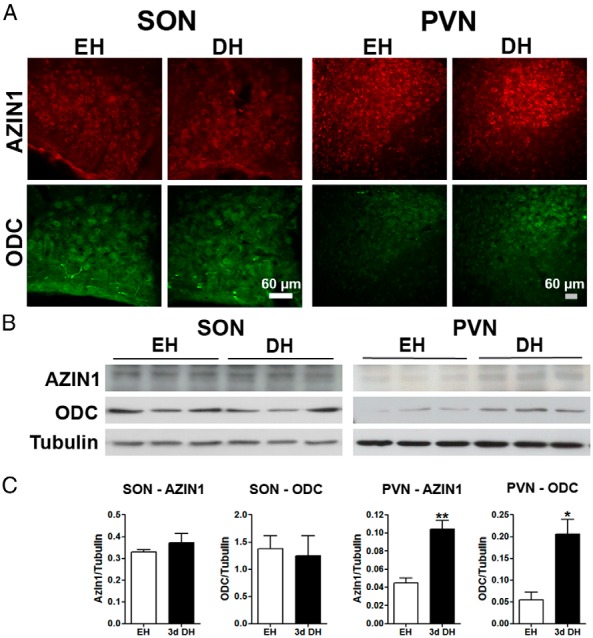
AZIN1 and ODC protein expression in SON and PVN of the EH and 3-day DH rat. A, Immunofluorescent localization of AZIN1 (red) and ODC (green) in EH and 3-day DH rats. B, AZIN1 and ODC protein expression was investigated by Western blotting in total protein extracts of SON and PVN from EH and 3-day DH rats. β-TUBULIN was used as the internal control gene. C, Densitometric analysis of AZIN1 and ODC protein expression in EH and 3-day DH rats. Values are means ± SEM of n = 3 animals per group. *, *P* < .05; **, *P* < .01.

Immunofluorescence staining was performed to compare AZIN1 and ODC expression in SON and PVN of EH and 3-day DH rats (Figure 3). The distribution of staining was similar in SON and PVN EH and 3-day DH rats (Figure 3A). The intensity of AZIN1 and ODC staining appeared higher in the PVN of 3-day DH rats compared with EH rats (Figure 3A). We performed Western blots of total protein extracts from SON and PVN of EH and 3-day DH rats to quantitatively investigate AZIN1 and ODC protein expression (Figure 3B). Immunoblots identified bands of approximately 50 kDa for both proteins as previously reported (26–28). Densitometric analysis of AZIN1 and ODC band intensity in EH and 3-day DH PVN samples, using β-TUBULIN as the internal control, showed significantly higher levels (AZIN1, *P* = .006; ODC, *P* = .017) of both these proteins after 3-day DH (Figure 3C).

### Polyamine modulation of gene expression in hypothalamic organotypic cultures

**Figure 4.**
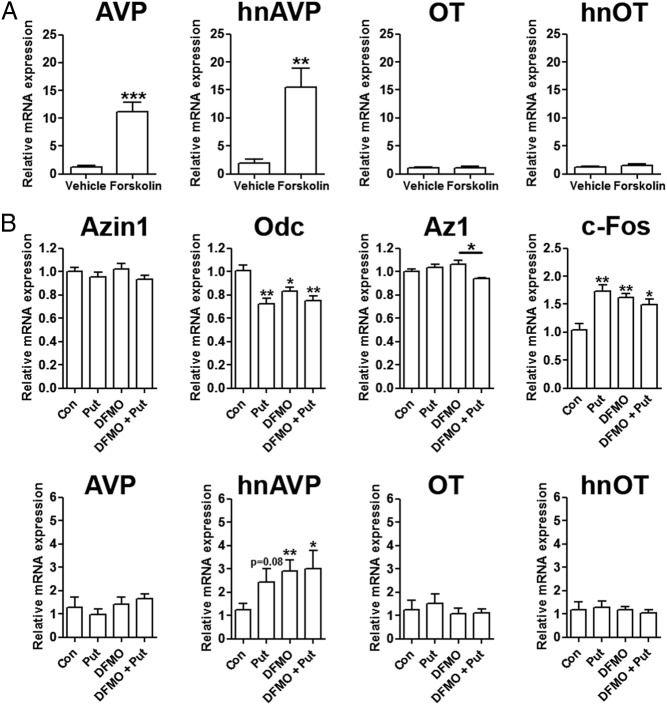
The differing effects of chemical treatments on gene expression in organotypic hypothalamic slice cultures. A, qPCR analysis of AVP, hnAVP, OT, and hnOT in PVN after 24 hours of treatment with 10 μM forskolin compared with vehicle (DMSO) controls. B, Relative mRNA expression of *Azin1*, *Odc*, *Az1*, *c-Fos*, AVP, hnAVP, OT, and hnOT was investigated after 48 hours of treatment with 10 μM putrescine (Put), 5 mM DFMO, and 5 mM DFMO and 10 μM Put compared with vehicle controls. Values are means ± SEM of n = 5 animals per group. *, *P* < .05; **, *P* < .01; ***, *P* < .001. Con, control.

We first tested our methodological approach by examining the expression of AVP in SON and PVN punches from organotypic slices after 24 hours of forskolin treatment compared with vehicle controls (Figure 4A). The expression of AVP was very consistent in each group (DMSO and forskolin) for PVN samples but varied greatly in each group for SON samples. Therefore, only PVN samples were used in our studies. AVP mRNA and heteronuclear RNA expression increased 10- (*P* = .0007) and 15-fold (*P* = .009), respectively, with no parallel changes in OT mRNA or hnOT expression, consistent with previous reports (29). We next asked whether the polyamine putrescine, or the irreversible ODC inhibitor DFMO, or a combination of both agents (to inhibit the DFMO effect) altered gene expression in punch samples from organotypic PVN slice cultures (Figure 4B). As a measure of the health of the culture system, we first analyzed *c-Fos* mRNA expression, which we found was increased by all treatments (putrescine, *P* = .003; DFMO, *P* = .004; DFMO + putrescine, *P* = .024), indicating an increase in neuronal activity (Figure 4B). There were no significant differences in *Azin1* mRNA expression across all treatments, whereas *Odc* mRNA expression was significantly reduced (putrescine, *P* = .002; DFMO, *P* = .023; DFMO + putrescine, *P* = .004) in all treatment groups compared with controls, (Figure 4B). *Az1* mRNA expression was unchanged by these treatments, except in cultures treated with a combination of DFMO and putrescine, in which we observed a significant decrease (*P* = .011) in expression compared with DFMO treatment alone (Figure 4B). Although the abundance of the mature AVP mRNA was not affected by these treatments, the expression of hnAVP, an indirect measure of AVP gene transcription, was significantly higher after the addition of DFMO (*P* = .01) and DFMO and putrescine (*P* = .036) to culture media compared with controls (Figure 4B). Neither putrescine, DMFO, nor a combination of both drugs affected OT mRNA or hnOT expression.

### Metabolic analysis after delivery of lentiviral *Azin1* shRNA into the SON and PVN

**Figure 5.**
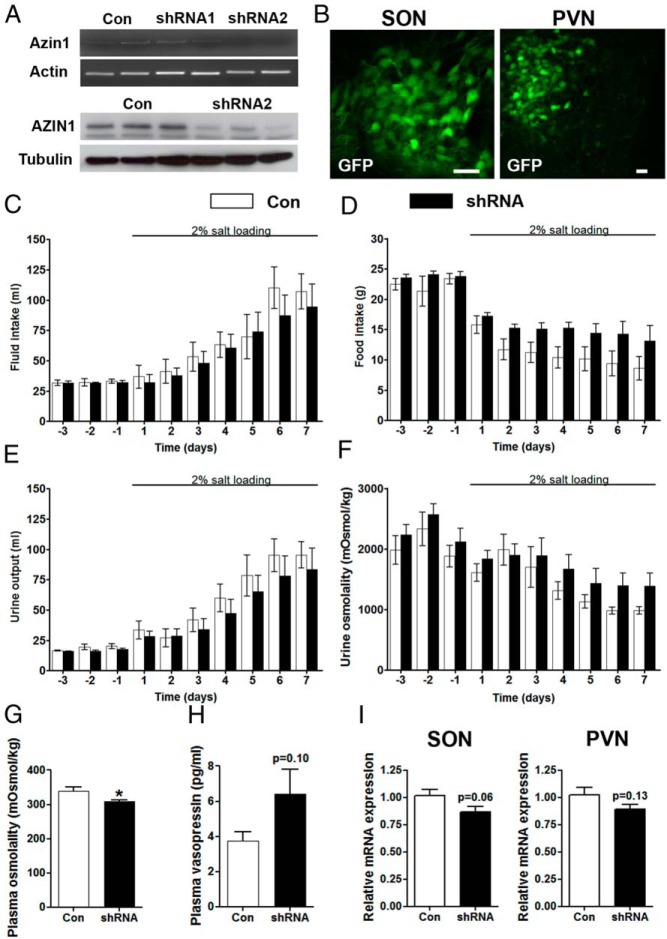
Metabolic analysis of gene function. A, RT-PCR and Western blotting validation of lentiviral-mediated knockdown of *Azin1* by shRNAs in rat PC12 cells. β-*Actin* was used as the internal control for RT-PCR and GAPDH for Western blotting. B, Lentiviral transduction of magnocellular neurons in the SON and PVN. Rats were bilaterally injected into SON and PVN with lentiviral vectors expressing *Azin1* shRNA2, or control shRNA. Two weeks after the injection, the rats were placed in metabolic cages for 10 days during which water intake (C), food intake (D), urine output (E), and urine osmolality (F) were recorded. On day 3 rats were presented with 2% (wt/vol) NaCl solution in place of drinking water for 7 days. Urine (F) and plasma (G) osmolality were measured by freezing point depression, and plasma AVP levels were determined by ELISA (H). I, Relative expression of *Azin1* in SON and PVN of *Azin1* shRNA-delivered animals compared with control shRNA. Values are means ± SEM of n = 6 animals per group. *, *P* < .05. Scale bars, 60 μm. Con, control.

We manipulated *Azin1* gene expression in the SON by shRNA-mediated knockdown. Of the two lentiviral Azin1 shRNAs we tested, shRNA2 showed significant knockdown of *Azin1* mRNA and protein expression in rat PC12 cells (Figure 5A). We thus delivered this shRNA2 into the SON and PVN of the hypothalamus by the stereotaxic surgical injection of lentiviral vectors. The neural tropism of these viruses was determined by analyzing GFP reporter expression in paraformaldehyde-fixed tissue (Figure 5B). We then proceeded to examine osmotic homeostasis in the transduced animals using metabolic cages. Rats injected with a nontargeting shRNA were used as controls (Figure 5, C–F). Basal measures of fluid intake (Figure 5C), food intake (Figure 5D), and urine output (Figure 5E) and urine osmolality (Figure 5F) under EH conditions were unchanged in *Azin1* shRNA-transduced animals compared with controls. As expected, a repeated-measures, two-way ANOVA showed that SL had a significant affect (*P* < .0001) on all metabolic measures in both the control shRNA and *Azin1* shRNA groups; fluid intake and urine output are increased, but food intake and urine osmolality are decreased. No significant effects of *Azin1* shRNA or control virus treatment were observed for fluid intake or any urine parameters. Interestingly, after the onset of SL, *Azin1* shRNA rats had a significantly higher food intake compared with the control shRNA rats (*P* < .0001) as calculated using a two-way ANOVA with Bonferroni post hoc test. The lower plasma osmolality (*P* = .042) observed in *Azin1* shRNA rats compared with control shRNA rats (Figure 5G) was not accompanied by significant effects on the plasma AVP levels (Figure 5H). Using qPCR, we observed lower *Azin1* expression (although not significant; SON, *P* = .06; PVN, *P* = .13) in animals injected with *Azin1* shRNA compared with the expression in control-injected nuclei after 7-day SL (Figure 5I).

### Lentiviral manipulation of *Azin1* and *Odc* expression in the SON results in altered AVP expression

**Figure 6.**
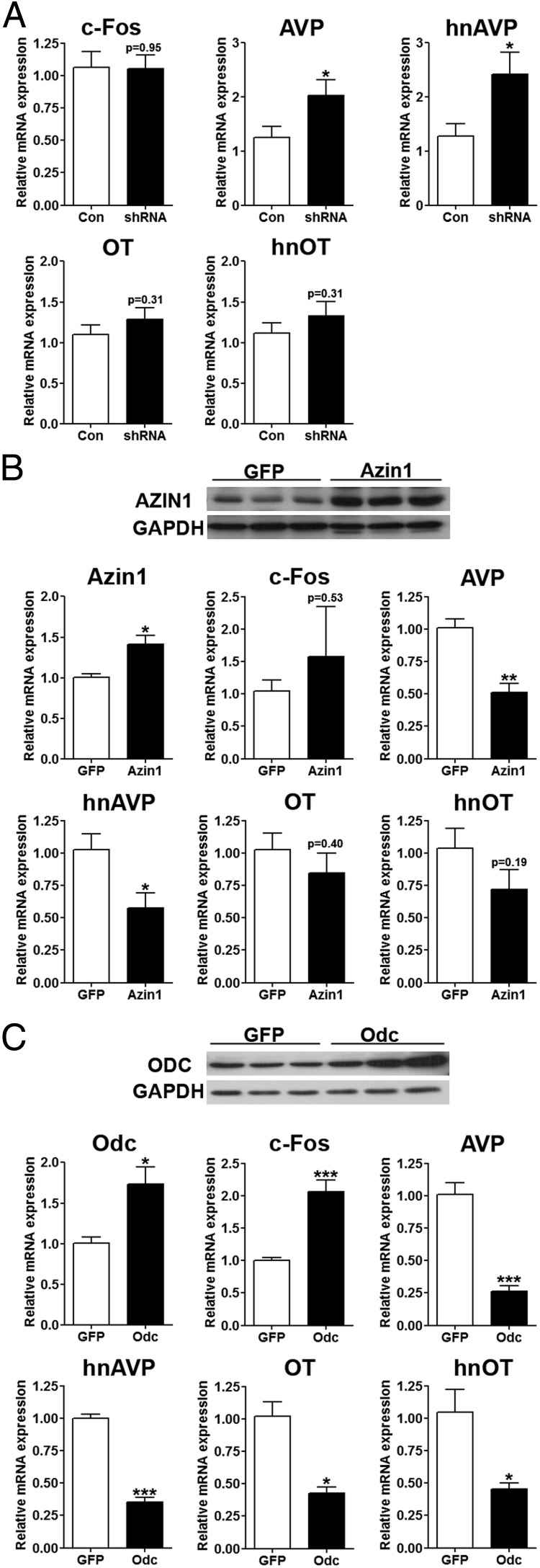
Lentiviral-mediated manipulation of *Azin1* and *Odc* expression in the SON alter AVP gene expression. A, Relative expression of *Odc*, *c-Fos*, AVP, hnAVP, OT, and hnOT in the SON of *Azin1* shRNA compared with control shRNA in rats subjected to metabolic analysis of 7-day SL (control shRNA, n = 11 SON; *Azin1* shRNA, n = 11 SON). Rats were unilaterally injected into SON with lentiviral vectors expressing rat *Azin1* and GFP (B) or rat *Odc* and GFP (C), and the expression of *Azin1*, *c-Fos*, AVP, hnAVP, OT, and hnOT was investigated 2 weeks later (GFP, n = 4 SON; *Azin1*, n = 4 SON; *Odc*, n = 4 SON). Inset (B, C) are Western blots of AtT20 cells (Sigma) transduced (5 multiplicity of infection) for 48 hours with lentiviral vectors expressing *Azin1* (B) or *Odc* (C) together with GFP controls. Values are means ± SEM. *, *P* < .05; **, *P* < .01; ***, *P* < .001.

The SON contains a largely homogenous population of MCNs compared with the PVN, which is more heterogeneous in nature, containing both magnocellular and parvocellular neurons. Therefore, to focus more specifically on overexpression/knockdown of *Azin1* and *Odc* (overexpression only) on gene expression in MCNs, the SON was chosen for these experiments. We examined gene expression in the SON of *Azin1* shRNA-injected knockdown rats compared with control shRNA rats (Figure 6A) and in *Azin1*- and *Odc*-overexpressing rats compared with GFP-transduced animals. Neither *Azin1* knockdown (Figure 6A), nor overexpression (Figure 6B), affected the expression of *c-Fos* mRNA, OT mRNA, or hnOT in the SON. Interestingly, we observed significantly higher AVP mRNA (*P* = .036) and heteronuclear RNA expression (*P* = .024) in SON injected with the *Azin1* shRNA compared with the control shRNA (Figure 6A). Furthermore, overexpression of *Azin1* (*P* = .015) had a negative effect on AVP transcription with significantly lower mRNA (*P* = .002) and heteronuclear RNA (*P* = .039) compared with GFP controls (Figure 6B). Overexpression of *Odc* (*P* = .016) reduced AVP (AVP, *P* = .0003; hnAVP, *P* = 7e^−06^) and also OT mRNA (*P* = .003) and heteronuclear RNA (*P* = .016) expression but increased the *c-Fos* mRNA (*P* = .001) expression in the SON (Figure 6C).

## Discussion

Polyamines are essential for cellular functions. Because polyamines possess only a limited capacity to cross the blood-brain barrier, their endogenous synthesis within the brain is necessary (30). We have previously reported up-regulated *Azin1* mRNA expression in the SON and PVN of 3-day DH male and female rats and in lactating rats (31, 32). In the present study, we have tested the hypothesis that an increase in *Azin1* expression may be important in the osmotic regulation of the expression of the two major neuropeptide products of the SON and PVN, namely AVP and OT.

We used qPCR to demonstrate robust increases in the expression of *Azin1* over a time course of DH, SL and after ip administration of HS, suggesting increased polyamine biosynthesis under these conditions. Indeed, previous studies have reported that overexpressing *Azin1* in cell cultures, and in the whole animal, increases polyamine synthesis (4, 33–37). Interestingly *Azin2*, which also increases polyamine biosynthesis by binding AZ to induce activation of ODC (38), was unaffected by DH and SL. The differing responses of *Azin1* and *Azin2* to hyperosmotic stress imply different cellular functions for these closely related genes as previously reported (38).

In contrast to the rapid increase *Azin1* expression, only SL increased *Odc* mRNA expression, with no effects seen on steady-state *Az1* mRNA expression. The physiological differences between DH and SL may underlie the differing responses. During DH, extracellular and intracellular fluid volumes decrease as water and sodium is lost in sweat and urine, resulting in hypovolemia and, as a consequence of DH-induced natriuresis, sodium depletion (39). In contrast, hypertonic saline drinking increases body sodium content, so increasing extracellular fluid volume whereas intracellular fluid volume decreases (39). In many systems, an increase in *Azin1* expression has commonly been shown to precede changes in *Odc* mRNA expression, similar to the changes presented here (4, 40). This may be explained by the complex interplay between these three regulatory components, which means that changes in *Odc* or *Az1* mRNA expression are not necessary to increase intracellular polyamines, which can be achieved by *Azin1* action alone (41). Therefore, in the early stages of hypertonic stress, it would appear that increased *Azin1* expression is required to maintain the rate of polyamine synthesis, whereas prolonging this stressor necessitates increased levels of *Odc* as well. Interestingly in the mouse, we have previously shown that DH results in *Odc*, not *Azin1*, up-regulation, suggesting species-specific alterations of genes in this pathway (32). One common feature of hypertonic stress is the increased transcription of AVP, as we have previously reported for DH and SL (21) and as we present here after HS. Interestingly, the increase of hnAVP transcription in SON and PVN after acute HS administration precedes any changes in *Azin1* transcription, suggesting that transcriptional changes in *Azin1* are not necessary for the initial response to these stimuli.

Immnocytochemical investigation revealed that AZIN1, ODC, AZ1, and spermine/spermidine are expressed in neurones positive for AVP- and OT-like staining. The expression patterns of ODC and spermine/spermidine immunofluorescence in the MCNs of SON and MCNs and parvocellular neurons of the PVN are similar to earlier reports in rats (7, 8, 42). Furthermore, the subcellular distribution of AZIN1, ODC, AZ1, and spermidine/spermine are consistent with previous in vitro and in vivo findings (7, 8, 42–46). The strong expression of AZ1 in the nucleus, as opposed to the strong cytoplasmic localization of its regulator AZIN1 in MCNs, may imply different subcellular levels of polyamine regulation in the cells. Indeed, Gritli-Linde et al (43) suggested that AZ1 proteosomal regulation of ODC may occur not only in the cytoplasm but also within the nucleus.

We then asked whether the increased *Azin1* mRNA observed in 3-day DH rats is translated into higher levels of the functional protein. The increase in *Azin1* mRNA corresponded with increased protein expression in PVN, but not SON, as shown by immunoblotting. In parallel, higher ODC protein expression was observed in the PVN but again not the SON. The detection of increased AZIN1 and ODC protein expression in the PVN, but not SON, may perhaps be explained by the heterogenous structure (paravocellular and MCNs) of the PVN relative to the largely homogenous structure of the SON. In agreement, elevated polyamines have been reported in the hypothalamus after acute and chronic restraint stress in rodents (47), suggesting that stress may perhaps alter polyamine synthesis in parvocellular components of the PVN. Another possibility is the stability of both AZIN1 and ODC proteins, which have extremely short half-lives (48–51), perhaps limiting measures at the protein level. Because the binding of AZIN1 to AZ1 prevents ODC degradation, stabilizing protein levels, an increase in AZIN1 levels may increase the abundance of ODC, as we observe in the PVN, resulting in elevated polyamine synthesis (41).

As expected, organotypic cultures responded to forskolin treatment with significant increases in AVP and hnAVP transcript levels (52). Treatment of these cultures with putrescine, DFMO, or a combination of both for 48 hours decreased *Odc* mRNA expression, but not *Azin1* or *Az1*, compared with controls. This decrease in *Odc* mRNA expression in groups treated with putrescine may have resulted as consequence of the negative feedback of this polyamine on ODC activity and gene expression (53). However, paradoxically, DFMO treatment also decreased *Odc* expression, although to a lesser extent, which may perhaps indicate increased compensatory polyamine intake, as previously reported (54, 55). To focus specifically on activity of MCNs and parvocellular neurons, we analyzed the expression of the AVP and OT genes. Of these neuropeptide transcripts, only hnAVP was altered, with increased expression observed after treatment with DFMO and DFMO with putrescine. The expression of hnAVP has previously been shown to be a sensitive indirect measure of the transcription of the *AVP* gene (56). We were surprised that the inhibition of ODC increased hnAVP expression because *Azin1* expression increases in parallel with AVP and hnAVP expression after DH and SL and HS (hnAVP only) (21). To explain these observations, we speculated that intracellular polyamines may in fact be negative regulators AVP expression and proceeded to test this hypothesis.

We used lentiviral vector delivery into the SON and PVN to either knock down endogenous *Azin1* expression using a specific shRNA or to overexpress *Azin1* or *Odc*. Although viral shRNA delivery mediated the efficient *Azin1* knockdown in vitro, this did not reach significance in vivo. This is possibly due to the inability to transfect MCNs throughout the SON using this methodological approach, compared with the high transfection efficiencies achievable using in vitro systems. Furthermore, shRNA-mediated gene knockdown has been shown to vary markedly across cell types (57), making it impossible to equate the level of knockdown in vitro with what is achievable specifically in MCNs. Nonetheless, *Azin1* shRNA delivery into the SON and PVN decreased plasma osmolality compared with control SL rats and had a statistically significant effect on food intake after 7-day SL. The lower plasma osmolality is supportive of a role for *Azin1* in fluid homeostasis. The increased food intake may underlie changes specifically within the PVN, which produces anorexigenic peptides such as thyrotropin-releasing hormone, CRH, and OT (58), or simply may reflect the increased hydration status of *Azin1* shRNA animals, as indicated by the lower plasma osmolality. Interestingly, spermine has been shown to suppress food intake when administered intracerebroventricularly into the rat brain, in agreement with a role of polyamines on ingestive behavior (59).

Plasma AVP levels appeared higher, although not significantly, in *Azin1* shRNA animals, possibly suggesting increased secretion, consistent with the lower plasma osmolality. Polyamines have been reported to regulate hormone secretion, and our data hint that this may be the case in AVP MCNs (60–63). Indeed polyamines have been shown to inhibit AVP release in neurohypophysis cultures, implying a role in the modulation of AVP release (64). Importantly, AVP mRNA and hnAVP mRNA expression was significantly increased in the SON after *Azin1* shRNA delivery, which is consistent with the trend toward an increase in peptide secretion. In contrast, *Azin1* overexpression in the SON resulted in decreased AVP mRNA and hnAVP expression. Therefore, we propose that increased *Azin1* expression, and by inference polyamines, in AVP MCNs act to negatively feed back to inhibit AVP synthesis brought about by DH and SL. Because polyamines regulate a range of cellular processes, it is not known whether increased AVP synthesis is responsible for increased *Azin1* expression during hypertonic stress. It is possible that *Azin1* expression is increased via activation of cellular mechanisms, in parallel but not directly, in response to increased AVP synthesis during hyperosmotic stress. The mechanisms regulating *Azin1* expression in MCNs during hyperosmotic stress remain to be determined.

We report here a rapid increase in hnAVP transcription in SON and PVN up to 1 hour after HS administration, with levels stabilizing thereafter consistent with the presence of inhibitory inputs to stabilize AVP gene expression. In agreement with this negative feedback hypothesis, previous studies have described increased nitric oxide synthase 1 expression in the SON of the DH rat (65). Inhibition of nitric oxide synthase 1 increases the secretion of AVP from MCNs, suggesting that nitric oxide production restricts peripheral hormone secretion in response to osmotic stimulation (66). These effects are thought to arise from nitric oxide inhibition of the firing of MCNs, representing an inhibitory feedback mechanism to regulate MCN activity. Any relationship with *Azin1* expression in the HNS, and the modulation of polyamine synthesis, remains to be determined.

OT gene expression was not significantly altered by the knockdown of *Azin1* or overexpression of *Azin1*. This was interesting, considering our observations of regulatory components of polyamine synthesis in both cell types, and because both AVP and OT synthesis in the SON and PVN is increased by both DH and SL (21, 67). Notably, OT expression appeared higher after knockdown and lower after overexpression of *Azin1* similar to AVP expression, suggesting that polyamines may also be important for OT expression. Moreover, *Azin1* is also increased in the SON and PVN by lactation (31), but it is not known whether knockdown or overexpression of *Azin1* gene would alter OT expression under these circumstances.

To strengthen our findings, we overexpressed *Odc* in the SON. The decrease in AVP and hnAVP expression by both *Azin1* and *Odc* overexpression suggests that these effects are mediated through changes in polyamine pathways. In addition, the observed decrease in OT mRNA and hnOT expression supports a further role for polyamines in OT transcription. An increase of *c-Fos* expression has previously been reported to coincide with increased ODC activity as a result of elevated intracellular putrescine content in rat kidney cells, consistent with our findings after the overexpression of *Odc* in the SON (68). Interestingly, the expression *c-Fos* was unaltered by *Azin1* overexpression, in which higher ODC expression would also be expected because ODC is rescued from targeted proteolytic degradation by AZ1 (4). These data perhaps suggest that higher levels of ODC, and therefore polyamines, are produced by *Odc* compared with *Azin1* overexpression. This may perhaps explain the larger decrease in AVP expression and significant decrease in OT expression resulting from *Odc* overexpression in the SON. Thus, our data strongly suggest that *Azin1*, by increasing polyamine levels in the SON, inhibits AVP gene expression. This is possibly at the level of transcription because we see changes in hnAVP levels, which is an indirect measure of AVP gene transcription (56). Indeed, polyamines have been shown to enhance and inhibit the DNA binding activity of transcription factors and to alter transcription factor expression (69–71).

In summary, our results showed that AZIN1 is highly expressed in vasopressinergic and oxytocinergic neurons of the SON and PVN, wherein expression is rapidly and robustly increased in response to various acute and chronic hypertonic stressors. Our data suggest that AZIN1, and by inference polyamines, are involved in the regulation of the expression of the AVP. We thus propose that AZIN1 is the component of the mechanisms involved in fine-tuning the levels of polyamines required in these hypothalamic nuclei to efficiently respond to physiological challenges.
